# Contribution of hurricane-induced sediment resuspension to coastal oxygen dynamics

**DOI:** 10.1038/s41598-018-33640-3

**Published:** 2018-10-24

**Authors:** Laura Bianucci, Karthik Balaguru, Richard W. Smith, L. Ruby Leung, Julia M. Moriarty

**Affiliations:** 1 Coastal Sciences Division, Pacific Northwest National Laboratory, Seattle, Washington, USA; 2 Global Aquatic Research LLC, Sodus, New York, USA; 3 Atmospheric Sciences and Global Change Division, Pacific Northwest National Laboratory, Richland, Washington, USA; 4 U.S. Geological Survey, Woods Hole Coastal and Marine Science Center, Woods Hole, Massachusetts, USA; 5 Present Address: Institute of Ocean Sciences, Fisheries and Oceans Canada, Sidney, British Columbia Canada

## Abstract

Hurricanes passing over the ocean can mix the water column down to great depths and resuspend massive volumes of sediments on the continental shelves. Consequently, organic carbon and reduced inorganic compounds associated with these sediments can be resuspended from anaerobic portions of the seabed and re-exposed to dissolved oxygen (DO) in the water column. This process can drive DO consumption as sediments become oxidized. Previous studies have investigated the effect of hurricanes on DO in different coastal regions of the world, highlighting the alleviation of hypoxic conditions by extreme winds, which drive vertical mixing and re-aeration of the water column. However, the effect of hurricane-induced resuspended sediments on DO has been neglected. Here, using a diverse suite of datasets for the northern Gulf of Mexico, we find that in the few days after a hurricane passage, decomposition of resuspended shelf sediments consumes up to a fifth of the DO added to the bottom of the water column during vertical mixing. Despite uncertainty in this value, we highlight the potential significance of this mechanism for DO dynamics. Overall, sediment resuspension likely occurs over all continental shelves affected by tropical cyclones, potentially impacting global cycles of marine DO and carbon.

## Introduction

Hurricanes, generically known as tropical cyclones (TCs), impact many coastal regions of the world by bringing strong winds and rain to both the land and sea. These extreme meteorological conditions affect many aspects of ocean circulation and land-ocean exchange, for example, by increasing wind-induced mixing and river discharge^1–3^. These physical changes also alter biogeochemical cycles in the coastal ocean. For instance, the ventilation and consequent re-aeration of the water column induced by hurricanes on the shelf has been linked to the smaller-than-predicted sizes of the Gulf of Mexico’s summer hypoxic region, also known as the “dead zone”^4–6^. However, other TC-induced processes could enhance DO consumption in the Gulf of Mexico as well as other continental shelves. Storm-enhanced rates of erosion in river basins and along the coastline lead to higher particulate^7–9^ and dissolved^10^ organic carbon loading to the shelf, which can result in increased biochemical oxygen demand. In some estuarine systems, the higher freshwater discharge and organic carbon input due to hurricanes lead to hypoxic (low DO) events^2,11–15^. Furthermore, extreme precipitation events in the Mississippi River basin were shown to rapidly transport terrestrially derived dissolved organic carbon to the northern Gulf of Mexico^16^. As a large portion of the terrestrial organic carbon is remineralized after it reaches the coastal ocean, the region changes from a net sink to a net source of carbon dioxide to the atmosphere during these events^16^. This flood-enhanced remineralization could also lead to additional consumption of DO.

In addition to the above-mentioned mechanisms by which TCs may affect DO, hurricane-induced resuspension may also increase DO consumption. Hurricanes have the necessary spatial scale and intensity to control continental shelf sediment dynamics, for example in the Gulf of Mexico^3,17–19^, the North American Atlantic coast^20^, and other parts of the world such as the high mountainous islands of the western Pacific^7,21^. The strong shear stress generated by hurricane-induced currents at the bottom boundary of the shallow continental shelf can resuspend sediments from as deep as the 100 m isobath^19,20^ and over spatial scales of kilometers^19^. Many studies have documented the massive volumes of sediment resuspended, transported, and re-deposited on continental shelves by TCs in different regions of the world. For example, 1.16 10^15^ ± 1.56 10^14^ g of sediment were determined to be mobilized along the Texas/Louisiana shelf during Hurricanes Katrina and Rita in 2005, representing 10 years of sediment output by the Mississippi River^18^. Large volumes of sediment transport have also been directly observed or modeled during many other Hurricanes in the Gulf over the last several decades^22,23^, along the US Atlantic coast during Hurricanes such as Isabel^24^ and Sandy^25^, and along the Northeastern Australian coast during Cyclone Winifred^26^. Sediment resuspension re-exposes sedimentary organic carbon (of both marine and terrestrial origin) and dissolved reduced substances to DO, leading to extensive remineralization of these pools^27,28^. The latter process enhances the production of carbon dioxide and consumption of DO, especially if the remineralization continues once the water column is re-stratified after the strong mixing event. The enhancement of DO consumption due to sediment resuspension has been investigated in environments not affected by hurricanes (e.g., Gulf of Finland^29^, Gulf of Lion^30^, a Scottish fjord^31^, a shallow U.S. lake^32^), using methods that range from benthic chambers with artificial stirring^29^ to numerical models^30^. A recent modelling study has also investigated this process in the Gulf of Mexico during non-hurricane conditions^33,34^. Furthermore, there is evidence in the Gulf of Mexico that sediments resuspended by a short-lived weather front led to increased oxygen utilization^35^. Nevertheless, the effect of hurricane-induced sediment resuspension on DO concentrations has not yet been thoroughly investigated.

In the present study, we aim to further the understanding of the role of sediment resuspension by hurricanes in coastal DO dynamics. Using a suite of datasets for the northern Gulf of Mexico including remote sensing, *in situ* observations, and reanalysis, we separate the effects on DO concentrations of hurricane-induced vertical mixing, the Mississippi/Atchafalaya River plumes, and resuspended shelf sediments. We find that within days of the hurricanes, the consumption of DO from sediment resuspension partly compensates for the effects of vertical mixing and is a significant sink of DO. While our focus is on the northern Gulf of Mexico, we argue that this process occurs on other continental shelves affected by extreme wind events and could be important for DO and carbon budgets of the global coastal ocean.

## Sediment Resuspension by Hurricanes

**Figure 1:**
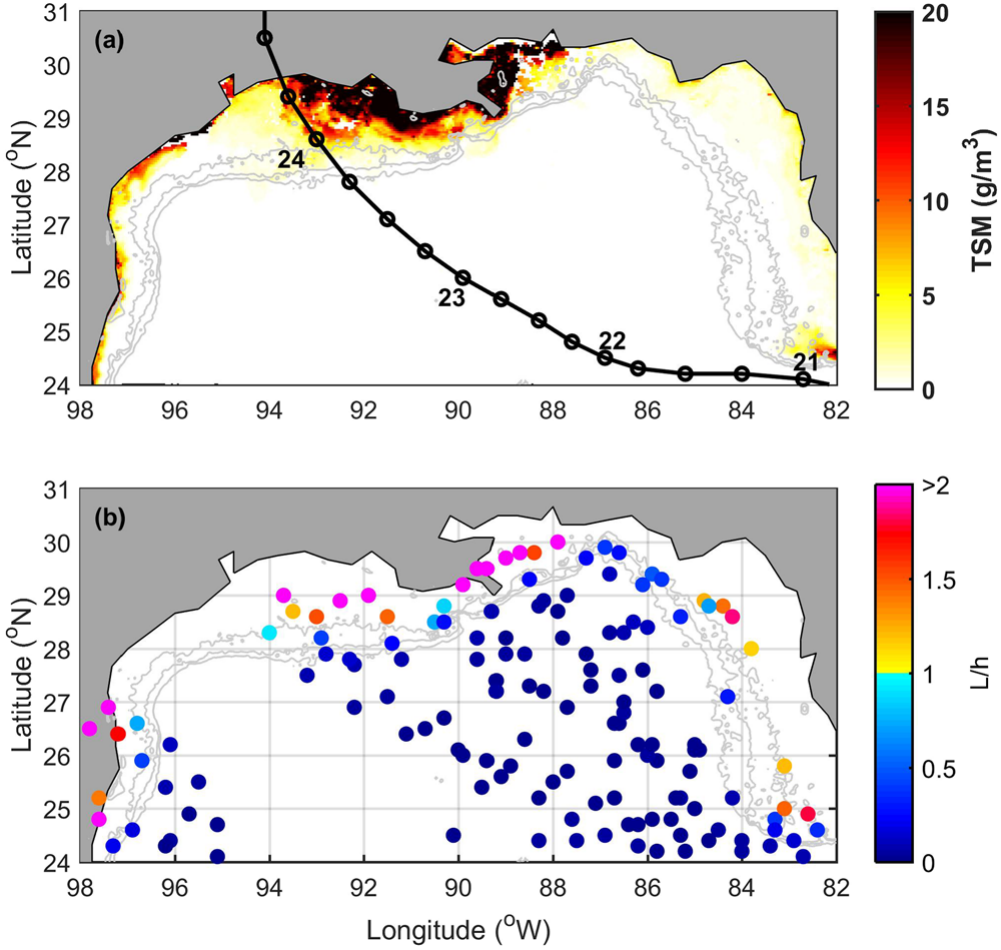
Maps of sediment resuspension. (**a**) Weekly total suspended matter (TSM) at surface in the Gulf of Mexico for the week in which Hurricane Rita made landfall (22 to 29 September 2005) and Rita’s track (numbers indicate day of September at midnight). (**b**) Proxy for sediment resuspension, L/h, where h is the bathymetry and L is the mixing scale calculated for hurricanes (wind speed > 32 m/s). The 50, 100, and 200 m isobaths are shown as gray contours in both panels. Maps were created using MATLAB (R2014a).

**Figure 2:**
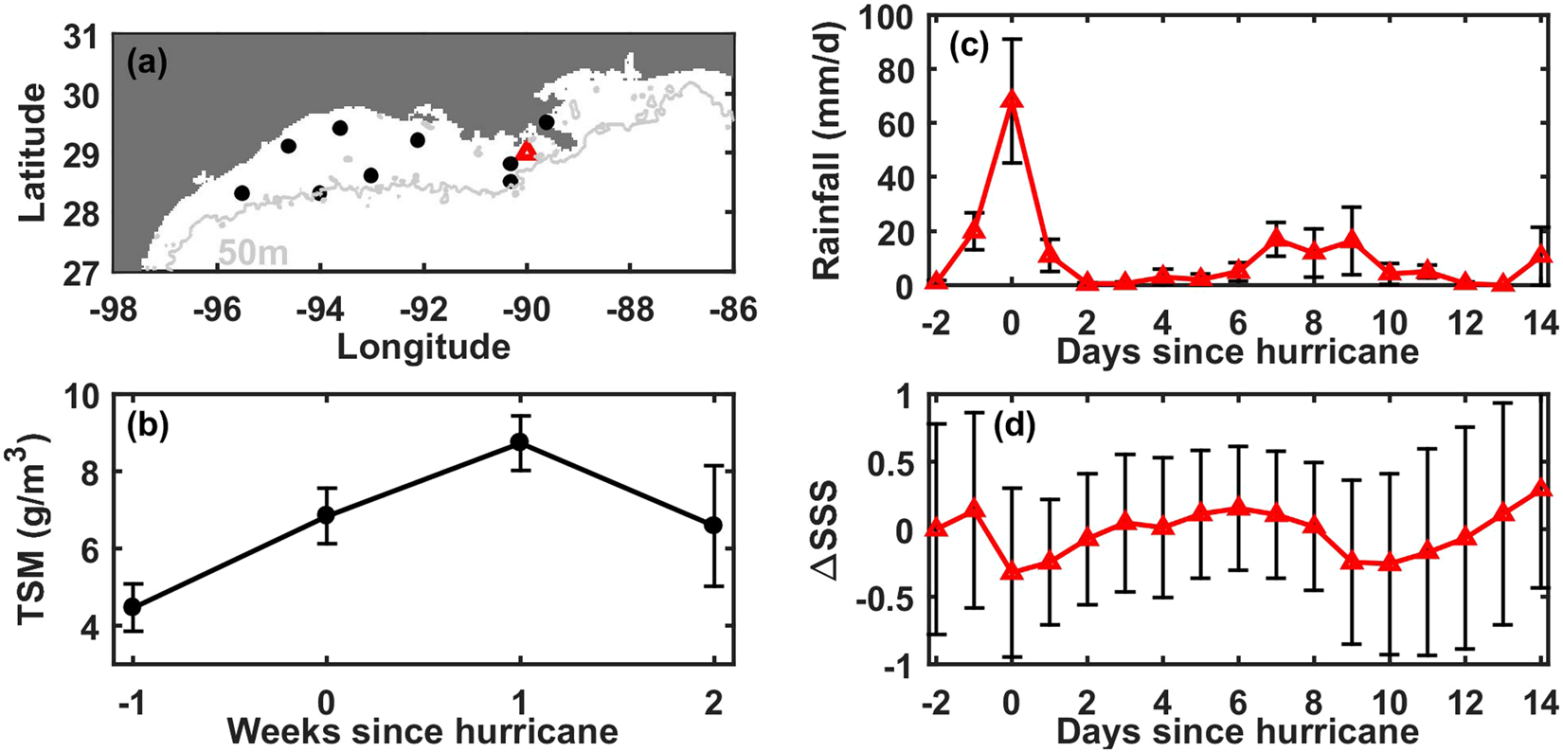
Total Suspended Matter (TSM), rain, and surface salinity composites. (**a**) Location of hurricane tracks in the northwestern Gulf of Mexico used to compute the TSM composites (black circles) and location of the station used to calculate the rain and salinity composites (red triangle). Map was created using MATLAB (R2014a). (**b**) Composite of weekly TSM (g/m^3^) from a week before to two weeks after the hurricanes. (**c**) Composite of daily rainfall (mm/d) and (**d**) sea surface salinity (SSS) from 2 days before to 14 days after the hurricanes. ΔSSS is shown by subtracting to SSS the value at day −2. Error bars in each composite show standard error.

As a measure of sediments resuspended from the ocean floor when hurricanes pass through the shelf, we used weekly satellite-derived Total Suspended Matter (TSM) concentrations (see Methods). For instance, a map of TSM for the Gulf of Mexico showed high concentrations the week Hurricane Rita made landfall (24 September 2005; Fig. 1a); TSM was highest landward of the 50 m isobath over the shelves of Texas, Louisiana, and Mississippi. In order to explore the spatial extent of potential hurricane-induced resuspension in the Gulf of Mexico, we developed a proxy for resuspension using a mixing length (L) associated with TC-induced wind forcing^36^ and the bathymetric depth (h). Resuspension would occur if L/h > 1, i.e. if the mixing generated by the hurricane reached the seafloor. Using available hurricane tracks from the HURDAT2 dataset and the corresponding density profiles at the location of each track from 1985 to 2010 from the Simple Ocean Data Assimilation (SODA) ocean reanalysis, we calculated L (eq. 1 in Balaguru *et al*.; more details about the equation, HURDAT2 and SODA in Methods) and then divided it by the bathymetry^37^. Values of L/h were usually above 1 in regions shallower than 50 m (Fig. 1b; although some studies suggest resuspension may occur up to 100 m depth during very strong storms^19,24^). For the locations of hurricane tracks within the 50 m isobath (Fig. 2a), we found the corresponding TSM for the week of the hurricanes, a week before the hurricanes, and up to two weeks after (more details in Methods). With these TSM values, we averaged the available data in each hurricane-referenced week and created a composite of TSM for the shelf region (depth < 50 m) of the Gulf of Mexico (Fig. 2b). A large increase in surface TSM (2.4 g/m^3^ or 53%) occurred the week of the hurricanes (“week 0”); during the following week (“week 1”), TSM further increased by an additional 42% (1.9 g/m^3^). By the subsequent week (“week 2”), TSM concentrations declined due to settling and nearly approached their pre-storm concentrations.

There are two main sources of surface TSM over the shelf during or after a hurricane: the sediments resuspended from the seafloor by hurricane-generated bottom shear^38,39^ and the additional suspended matter carried into the region by freshwater discharge from the Mississippi/Atchafalaya river system and several smaller rivers along the coastline (all of the above may integrate additional TSM from the erosion of wetlands). In order to distinguish between the timing and magnitude of these two sediment sources in our TSM composite, we analyzed the rain and sea surface salinity (SSS) signatures near the mouth of the Mississippi River at 29°N and 90°W (red triangle in Fig. 2a; see details on rain and SSS data in Methods). For example, SSS and rainfall in late summer/early fall 2005 showed two freshening events after each of the hurricanes Katrina and Rita: the first one was an immediate response to rainfall and the second lagged for ~5–7 days, indicating the arrival of the river plume (Supplementary Fig. S1). To generalize these findings to all hurricanes, we followed a similar approach as for TSM and created composites of SSS and rainfall from two days before the arrival of hurricanes to 14 days after (Fig. 2c for rainfall and Fig. 2d for ΔSSS, which is SSS minus SSS two days before the hurricanes). This analysis showed a mean decrease in SSS around day 0, followed by a second freshening around days 9 and 10 after the hurricanes (Fig. 2d), consistent with our findings for Hurricane Katrina and Rita (Fig. S1). Given the large amount of precipitation during the hurricanes relative to the change in SSS (Fig. 2c), as well as analysis of Mississippi River hydrographs during several Gulf of Mexico storms showing that the typical residence time of freshwater in the watershed and lower River is ~2 weeks (see Supplementary Material and Fig. S2), we assumed that rainfall and shelf processes (rather than changes in riverine input) primarily drove changes in SSS and surface TSM within the first 5–7 days after the storms. In contrast, the second instance of freshening was likely due to the re-establishment of the river plume following the storm (to a lesser extent, rainfall on the shelf may also have contributed to the second decrease in salinity). Based on the above and given that the ΔSSS composites represent an average response of SSS to hurricanes at a location close to the Mississippi River mouth (i.e., the plume will take longer to arrive to the northwestern shelf, even if winds and other factors affect the plume location), we assumed that riverine delivery has minimal effect on the shelf TSM in surface waters within the first ~5–7 days after the storms.

## Connection Between DO and Resuspended Sediments

**Figure 3:**
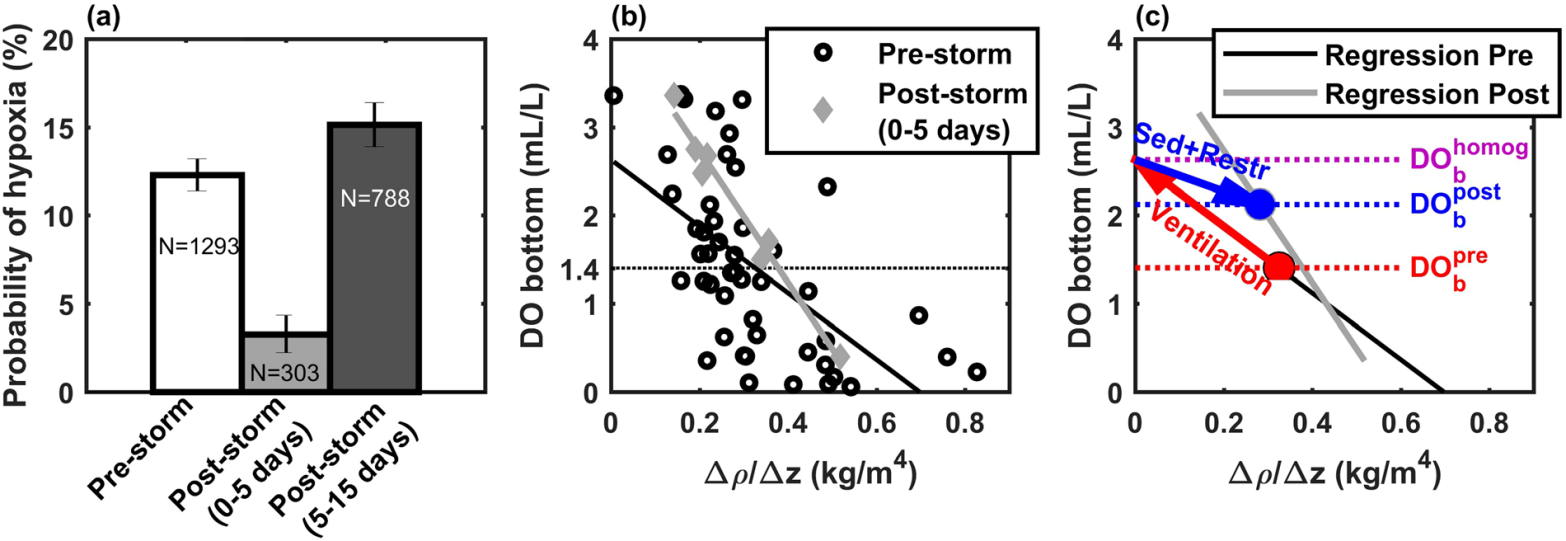
Analysis of *in situ* dissolved oxygen (DO) data. (**a**) Probability distribution function of hypoxia (DO ≤ 1.4 mL/L) for three periods: 2 weeks before, 0 to 5 days after, and 5 to 15 days after hurricanes (error bars show standard deviations and each column shows sample sizes). (**b**) Bottom DO vs. stratification (Δρ/Δz) for the pre-storm (open black circles) and 0 to 5 days post-storm (filled gray diamonds) periods. Regression lines are shown for each period. (**c**) Schematic for 2-step DO analysis. Red and blue circles indicate the mean DO conditions pre- and post-hurricane, respectively. Arrows show the changes in DO due to complete homogenization (ΔDO_ventilation_, red arrow) and the combination of restratification and remineralization of resuspended sediments (ΔDO_sedim+restrat_, blue arrow). Horizontal dotted lines highlight the values of bottom DO for the pre-storm, post-storm, and fully-homogenized conditions.

While surface sediment concentrations do not equal (and likely underestimate) near-bed concentrations, the resuspension-induced signal in surface TSM implies that sediment concentrations increase throughout the water column, including near the bed. The aerobic remineralization of the organic carbon in these near-bed sediments could represent a significant DO sink for bottom waters. In order to investigate changes in bottom DO due to hurricane-induced sediment resuspension, we used an extensive dataset of *in situ* DO observations in the northwestern Gulf of Mexico (see Methods and Supplementary Material). We selected stations located at depths shallower than 50 m, within 200 km of a hurricane track, and within three different time periods relative to the hurricane: 2 weeks before, 0 to 5 days after, and 5 to 15 days after the hurricane (note that no observations were available in the 5 days right before and 2 days right after hurricanes). The probability distribution functions of hypoxic waters (DO ≤ 1.4 mL/L) show how hypoxia decreased considerably right after the hurricane and rebounded back to previous conditions shortly after (Fig. 3a), which is consistent with previous studies^4,5,40^. These studies focused on the role of water column re-aeration to explain the decrease in hypoxia shortly after the hurricanes. Given the observed increase in surface TSM during the week of the hurricane (which we linked to sediment resuspension affecting the whole water column), here we propose instead that two main processes were at play between the pre-storm and the post-storm (0 to 5 days after hurricanes) conditions: (1) the DO replenishment due to mixing/ventilation of the water column; and (2) the DO consumption by the remineralization of resuspended sediments (which are mostly composed of terrigenous clay and silt in the region of our DO observations^41^, with an organic content of up to 4.4%^35^). The latter process was also aided by some re-stratification, which reduced the DO supply to the bottom waters and concentrated the resuspended organic matter in a smaller volume; re-stratification was likely due to post-storm solar heating, flow from small, flashy rivers and an initial pulse from the lower Mississippi/Atchafalaya system.

In order to distinguish between the two proposed processes, we analyzed bottom DO concentrations (DO_b_) vs. stratification for the two periods (Fig. 3b). Previous studies showed that the strength of vertical stratification is an important predictor of DO_b_ in the Gulf of Mexico^40,42^. However, the effect of stratification on DO_b_ may increase as the rate of DO consumption is enhanced, e.g. due to the availability of additional organic matter. As a measure of stratification, we used the difference in density between bottom and surface samples divided by depth: Δρ/Δz = (ρ_bottom_ − ρ_surface_)/h. We chose to regress DO_b_ as function of Δρ/Δz with an ordinary least square fit (see Methods); nevertheless, the use of other regression methods did not affect our final conclusions significantly (see Supplementary Material and Table S1). We found that DO_b_ had statistically different (p < 0.0001) regressions for the pre- and post-hurricane periods:

1 $$D{O}_{{b}}^{{pre}}=-\,3.77\cdot {({\rm{\Delta }}\rho /{\rm{\Delta }}z)}^{{pre}}+{\rm{2.6}}$$

2 $$D{O}_{b}^{post}=-\,7.49\cdot {({\rm{\Delta }}\rho /{\rm{\Delta }}z)}^{post}+4.2$$

where Δρ/Δz and DO_b_ have units of kg/m^4^ and mL/L, respectively (see more details on these equations in Methods). Overall, the slope for the post-storm period (−7.49 ± 0.55) was steeper than for the pre-storm conditions (−3.77 ± 0.73). Assuming that lateral fluxes of DO are small (see Supplementary Material), the change in slope indicates an enhancement in DO depletion right after the hurricanes; more precisely, the almost doubling of the regression slope indicates that DO depletion about doubled (99% higher, because 7.49/3.77 = 1.99 ± 0.08) in the days after the storm, compared to pre-storm conditions. We suggest that this is due to the resuspended sediments in the water column and the associated increase in the remineralization of their organic content.

To quantify the role of remineralization of resuspended sediments vs. that of ventilation of the water column, we considered DO dynamics during the passage of a hurricane that completely homogenizes the water column. This assumption of vertical homogenization was justified by analyzing HYCOM data before and after hurricanes, which showed that hurricanes mostly homogenized the water column in the shallow continental shelf regions of the northern Gulf of Mexico (see Supplementary Material and Fig. S5). For this analysis, we propose that the effect of mixing/ventilation in DO_b_ may be represented by the changes in stratification following equation (1), while equation (2) reflects the combined effect of re-stratification and resuspension. When assuming complete homogenization, the change in DO_b_ due only to ventilation (ΔDO_ventilation_) could be calculated as the difference between equation (1) applied to a) Δρ/Δz = 0 (homogenized water column) and b) the mean stratification for the pre-storm period (Δρ/Δz = 0.33 kg/m^4^; note that DO_b_^pre^(Δρ/Δz = 0.33) = 1.4 mL/L is equivalent to the observed mean DO_b_ concentration for the pre-storm period). The result is ΔDO_ventilation_ = 2.6–1.4 = 1.2 ± 0.2 mL/L (see red arrow in Fig. 3c and detailed calculations in Supplementary Material). After complete homogenization of the water column, the remineralization of resuspended sediments and the associated increase in DO consumption, as well as the reduction in DO supply due to re-stratification, decreased DO_b_ concentrations and led to the observed mean DO_b_^p^°^st^ of 2.1 mL/L (equivalent to equation (2) applied to the mean post-storm stratification, Δρ/Δz = 0.28 kg/m^4^). Therefore, the mean combined effect of sediment remineralization and re-stratification would be the difference between DO_b_^pre^ at homogeneous conditions (2.6 mL/L) and mean DO_b_^p^°^st^ (2.1 mL/L), yielding ΔDO_sedim+restrat_ = −0.5 ± 0.4 mL/L (blue arrow in Fig. 3c). The combination of ΔDO_ventilation_ and ΔDO_sedim+restrat_ (1.2 mL/L – 0.5 mL/L = 0.7 mL/L) accounts for the difference between the observed mean DO_b_^pre^ and DO_b_^p^°^st^ (i.e., 1.4 mL/L vs. 2.1 mL/L, respectively). Hence, by assuming an intermediate step with a completely homogenized water column, the combined effect of sediment remineralization and re-stratification represented 42% of the effect of re-aeration (0.5/1.2 = 0.42 ± 0.31). Here, we assumed a linear combination of both processes (i.e., ventilation and the combination of remineralization and re-stratification) because hurricane-induced vertical mixing is almost instantaneous compared with sediment remineralization and re-stratification. This 42% represents an upper limit for the role of resuspended sediments and re-stratification on bottom DO concentrations, because hurricanes may not necessarily homogenize the whole water column. However, our analysis of HYCOM data (see Supplementary Material) indicates that hurricanes (and even tropical storms) are typically able to create near-homogenous conditions in the water column. Furthermore, model results also suggest that remineralization of resuspended particulate organic matter (POM) is the major contributor to the storm-induced increase in DO consumption rate during and following Hurricane Humberto in 2007 (see Supplementary Material), consistent with Gulf of Mexico modeling results for smaller storms^33,34^.

We assumed that ΔDO_sedim+restrat_ is a linear combination of its “sediment-only” and “restratification-only” components due to the different time scales of remineralization and restratification (slow vs. fast, respectively). Revisiting the concept that the availability of resuspended sediments increases DO_b_ consumption by 99% for any given change in stratification, we then have ΔDO_sedim+restrat_ = ΔDO_sedim_ + ΔDO_restrat_ = 1.99 ΔDO_restrat_. Therefore, using the upper bound ΔDO_sedim+restrat_ = 0.42 ΔDO_ventilation_, we calculated that the effect in bottom DO by the remineralization of resuspended sediments (ΔDO_sedim_) represents up to 21 ± 15% of the re-aeration of the water column. Analogously, the re-stratification after the hurricane-induced mixing (ΔDO_restrat_) also represents up to a 21 ± 16% of the re-aeration. This result was consistent with estimates made using other linear regression techniques (e.g. ΔDO_sedim_ was a 12 ± 3% and 16 ± 6% of ΔDO_ventilation_ with two model II regressions, see Supplementary Material and Table S1).

There is high uncertainty in the above calculations (~±16%), but alternative methods of estimating the effects of resuspended organic matter on DO support our conclusion, as shown below. The uncertainty is due to the large scatter in the pre-hurricane dataset and the limited number of observations immediately post-hurricanes. Additional errors arise from using observations from different storms and different sampling locations for the pre- and post- storm observations. Eventually, future sampling programs should be able to reduce this uncertainty through measurements made closely before and after hurricanes; in the meantime, we offer results from a numerical model and an alternative back-of-the-envelope calculation to further support our results. For the latter, we aim to estimate how much DO would be consumed by the remineralization of the TSM that represents hurricane-resuspended sediments in our composite (6.85 g/m^3^ at week 0, Fig. 2b), keeping in mind that this surface TSM likely underestimates the near-bed sediment concentrations. To transform TSM into organic carbon (OC) units, we use two different OC content values from the Louisiana Shelf^ 35^: 4.4%OC measured for TSM in the nepheloid layer and 1.4%OC from seabed samples (the surface TSM measured by satellite should have an intermediate %OC, since hurricanes will bring up to the surface both deposited sediments and sediments already in suspension in the nepheloid layer). Using these values and considering a ratio of oxygen utilization to organic carbon consumption of 1.3^35^, the DO sink from the remineralization of the hurricane-resuspended surface TSM would range between 0.09 and 0.28 mL/L. This rough calculation is in reasonable agreement with our estimation of DO_b_ consumed by resuspended sediments (ΔDO_sedim_ = 0.21 × ΔDO_ventilation_ = 0.21 × 1.2 mL/L = 0.25 mL/L). Furthermore, results from a numerical model for the Gulf of Mexico suggest that decomposition of resuspended sediments could account for an even greater proportion of re-aeration (e.g. 56% in the bottom water column during Hurricane Humberto in 2007, see Supplementary Material).

Previous studies noted that the alleviation of hypoxia by hurricanes depends on many factors^4,43^, including the timing of the storms. For example, conditions in early summer, when wind stress decreases after the winter period^44^ and freshwater volume on the Texas – Louisiana shelf is high^45^, would allow for stratification and hypoxia to re-establish more easily after a hurricane. In contrast, several factors would affect the re-establishment of stratification and hypoxia after TCs later in the summer, e.g. the increased frequency of autumn/winter storms^43^, the reduced availability of freshwater over the shelf^45^, and the decreased availability of labile POM (which would lower DO consumption)^46,47^. Our results indicate that, on average and when considering as many hurricanes as we could match with our DO dataset (see Methods), hypoxia 5 to 15 days after a hurricane returned to the pre-storm levels (Fig. 3a). This re-establishment of hypoxia co-occurred with the arrival of the hurricane-enhanced river plume, the reappearance of strong stratification, and the further increase in TSM in the week after the hurricanes. Furthermore, the organic matter brought by the river plume is likely fresher than in shelf sediments, which have already undergone substantial oxic breakdown^48^. Further analysis of the available data for the 5–15 day period after hurricanes is given in the Supplementary Material.

## Implications of Sediment Resuspension by Hurricanes

While the ventilation and re-aeration of the water column is the dominant effect in bottom DO concentrations during or shortly after the passage of a hurricane in the northern Gulf of Mexico, here we show that the oxidation of organic matter associated with resuspended sediments partly opposes the effects of ventilation. This process has previously been neglected in the analysis of how hurricanes affect oxygen levels, but it provides a DO sink that weakens the alleviation of low DO conditions by wind-induced mixing. Previous studies have found that resuspension can decrease DO during non-hurricane conditions^29,49,50^; for instance, a recent modeling study showed that sediment resuspension increased DO consumption up to 8 times offshore of the Rhône River Estuary^30^. To the best of our knowledge, our work represents the first attempt at quantifying the role of hurricane-induced sediment resuspension in coastal DO dynamics.

Although the limited availability of observations creates uncertainty in our results, this work highlights the potential significance of the proposed mechanism. Future sampling programs aimed at measuring water column properties closely before and after TCs would reduce the uncertainty in our current estimates. Furthermore, targeted observations and numerical models may be able to address other processes that we could not resolve with our dataset, such as horizontal advection. For instance, model results show that the dominant drivers of DO during and shortly after Hurricane Humberto (2007) were vertical mixing/advection and the decomposition of hurricane-induced resuspended sediments, rather than horizontal advection (see Supplementary Material). Another process unaccounted for by our dataset is the enhancement of aerobic remineralization rates by the overall increase of DO by re-aeration; nevertheless, model results show that the role of this enhancement is minor compared to sediment resuspension^33,34^.

The Gulf of Mexico has the distinctive feature of a large river plume, but not every region affected by TCs counts with a major river. The effect of the Mississippi/Atchafalaya river plume was crucial for the re-establishment of hypoxia in the Texas – Louisiana shelf in our second post-storm period (5–15 days after the hurricanes); we could expect the increase of low DO waters following storms to be slower in shelf regions affected by hurricanes but unaffected by large river plumes. From a global perspective, only 15% (1.3 × 10^6^ km^2^) of the total shelf area affected by TCs in the world (8.5 × 10^6^ km^2^ of shelves shallower than 200 m) is impacted by major rivers (see Methods). In contrast, resuspension of previously accumulated sediments occurs over every shelf and coastal region affected by storms^24^, even in regions without large river systems^51^ (see Supplementary Fig. S10 for an example of high TSM after a hurricane on the Florida shelf). Furthermore, non-deltaic sediments often contain about double the organic carbon than deltaic sediments^52^, potentially amplifying DO drawdown because their bacterial breakdown consumes more DO. In some shallow estuarine systems where hypoxia developed shortly after the impact of hurricanes (attributed to the higher discharge and freshwater loading^2,11–15^), resuspended sediments could have also partly contributed to the drawdown of DO. In summary, the effect of DO consumption by the decomposition of resuspended sediments would be ubiquitous and could potentially have a role in global coastal DO and carbon dynamics. Moreover, the effect could be relatively more important in maintaining low DO values in shelf areas without large river plumes.

Besides furthering the understanding of DO dynamics in coastal regions affected by TCs, the role of sediment resuspension could have implications for model-based calculations of DO budgets. Many large-scale global and regional ocean models do not consider sediments explicitly^42,53–56^ and thus, ignore sediment resuspension as a mechanism affecting DO dynamics. Therefore, their budgets could be overestimating the re-oxygenation due to TCs as well as smaller ventilation events. Furthermore, given the tight connection between DO and carbon cycles, our results strengthen previous suggestions that sediment resuspension by TCs may have a quantitatively significant impact on coastal carbon cycling^28,57^.

The long-term DO decline observed in the global ocean is of concern^58–60^ due to the expected and potential negative effects to marine ecosystems, greenhouse gas emissions (particularly N_2_O), and consequently, to society. Hurricanes are traditionally expected to re-oxygenate the water column in the coastal ocean; however, here we described a TC-related mechanism that would partly counteract this re-aeration on continental shelves. As hurricane intensity is expected to increase under future climates^61,62^, it remains unclear whether the mechanism proposed here would increase or decrease its relative importance with respect to wind-induced ventilation. The relative role will depend, at least partly, on whether more sediments can be resuspended under stronger or more frequent hurricanes and whether ventilation would also decrease due to the effect of warmer future conditions in DO solubility. Overall, this study emphasizes the importance of efforts to understand the role of extreme events on ocean biogeochemistry, as the frequency and intensity of events such as hurricanes, floods, drought, etc. may change in the future.

## Methods

### Hurricane data and mixing length

Hurricane track data were obtained from the National Hurricane Center’s HURDAT2 database^63^ (www.nhc.noaa.gov/data/#hurdat) to calculate the composite mean sediment and sea surface salinity response to hurricanes, hurricane rainfall composite, and the hurricane mixing length (L). To compute the latter, we used a turbulent kinetic energy approach that considers the balance between work done by the wind at the surface and the potential energy barrier created by ocean stratification^64^; therefore, L is estimated based on the hurricane’s intensity, its forward moving speed, and the upper-ocean stratification beneath the storm^36^. This stratification was calculated from the Simple Ocean Data Assimilation (SODA) ocean reanalysis^65^, using 5-day mean, sub-surface temperature and salinity data at a spatial resolution of 0.5 degrees (obtained from www.atmos.umd.edu/~ocean/).

### Total suspended matter data

Weekly total suspended matter (TSM) from April 2002 to April 2012 at 4 km spatial resolution was obtained from the European Space Agency’s GlobColour Project^66,67^ (www.globcolour.info); these data have been developed, validated, and distributed by ACRI-ST, France. TSM is given in units of grams per cubic meter and is a measure of the turbidity of the water. It uses the MERIS C2R Neural Network algorithm^68^ and is computed from the back-scattering coefficient at 444 nm. The product is valid for case 2 waters, i.e. waters where inorganic particles dominate over phytoplankton (typically in coastal waters).

To compute the composites, we first found the hurricane tracks available in the Northern Gulf of Mexico (north of 28°N) within the 50 m isobath. Then, for each track we found all the weekly TSM data available within 2 degrees of the track location and with depths ≤ 50 m, which approximately represents the median radius of Atlantic hurricanes^69^. We assigned the name “week 0” to all the weeks of TSM data in which a hurricane track occurred within the first 5 days of the week, in order to assure that the response to the hurricane was captured by that week’s data. Weeks prior to or after “week 0” were indicated with negative or positive numbers (e.g., “week −1”, “week 1”). Finally, the data available for all hurricanes was averaged across weeks, creating the TSM composite (Fig. 3b).

### Rain and sea surface salinity data

Daily satellite precipitation data from NASA’s Tropical Rainfall Measuring Mission^70^ (https://pmm.nasa.gov/trmm) was obtained for the period 1995–2011 at a location near the mouth of the Mississippi River (90°W and 29°N). To compute the rainfall composite, we found each hurricane passing within 200 km of that location, finding all precipitation values from 2 days prior to the storm’s arrival to 14 days after the day of the storm. The composite was created by averaging the rainfall obtained for 7 hurricanes at each of those 17 days. The same procedure was followed to create the ΔSSS composites, using SSS at 90°W and 29°N from the 1998–2015 data-assimilative HYbrid Coordinate Ocean Model (HYCOM) Global Ocean Forecasting System 3.1 (https://hycom.org/)^71^ and removing the value of SSS two days before the hurricane to the time series to create ΔSSS. The standard error for ΔSSS is larger than for rain (error bars in Fig. 2d and c, respectively), because the changes in SSS due to precipitation are small compared with the seasonal cycle.

### Dissolved oxygen data

DO observations belong to the World Ocean Database 2009 (WOD09)^72,73^, the Mechanisms Controlling Hypoxia (MCH) program, and other published sources^4,6,74–77^. The whole dataset counted observations from 1933 to 2011, from which we selected stations that satisfied three criteria:

- Water depth shallower than or equal to 50 m, since TCs mostly resuspend sediments at these depths according to our L/h criteria (Fig. 1b).
- Location within 200 km of a hurricane track from the HURDAT2 dataset (as mentioned before, this distance represents the radius of influence of hurricanes in the Gulf of Mexico^69^).
- Timestamp within three different periods relative to a hurricane track: between 1 and 14 days before, between 0 and 5 days after, or between 5 and 15 days after.

The locations of selected stations as well as their corresponding DO histograms are shown in the Supplementary Material (Fig. S6). For the pre-hurricane period, we obtained 48 DO profiles that occurred before three hurricanes: Andrew (1992), Lili (2002), and Katrina (2005). None of these profiles were sampled in the 5 days right before the hurricanes; furthermore, only data for Katrina was available within 10 days of the storm (hence, our extension to 14 days in order to capture two more hurricanes). For the period 0–5 days after hurricanes, we had 7 profiles measured between 2 and 4 days after hurricanes Danny (1997) and Cindy (2005). Note that while observations from the before and 0–5 days after periods belong to different hurricanes, environmental conditions were typical of summer in all cases (i.e., pre- and post-storm profiles showed conditions within the range of observations unaffected by hurricanes at the same locations). Lastly, for the period 5–15 days after, we found 97 stations for 8 hurricanes: Babe (1977), Bonnie (1986), Andrew (1992), Danny (1997), Lili (2002), Cindy (2005), Katrina (2005), and Rita (2005). However, given that the spatial coverage for this period was so much broader than for the other two periods, we spatially subsampled these data to represent the same spatial area as the dataset for the period 0–5 days after the hurricanes. The subsampled dataset counts with 36 DO profiles matched for 6 hurricanes (same as for the full dataset except for hurricanes Babe and Rita).

The regressions in equations (1) and (2) were fitted with ordinary least squares (even if the independent variable Δρ/Δz likely has error associated to it), because the relationship between the two variables is expected to be asymmetric^78^, i.e. stratification is the proposed driver of DO_b_, while the opposite is not true. Nevertheless, the results using two other least square fits that do assume error in the “x” variable do not alter our main conclusions and are shown in the Supplementary Material and Table S1. To evaluate the calculated regressions between bottom DO and stratification in equations (1) and (2), we tested the null hypothesis on the Pearson product-moment correlation coefficient (R) with a t-test. For equation (1), R = −0.60 (R^2^ = 0.37), N = 48, t = −5.2 and degrees of freedom = 46. For equation (2), R = −0.99 (R^2^ = 0.97), N = 7, t = −13.6 and degrees of freedom = 5. Therefore, both R values were significant at more than 99% (p < 0.0001); note that the high R^2^ in equation (2) is due to regressing observations from only two cruises. We applied the Fisher r-to-z transformation to assess the significance of the difference between both R values; the result provided z = 3.46 and a difference significant at more than 99% (for either the one- or two-tailed cases, with p = 0.0001 and 0.0002, respectively). In addition, we tested that the slopes of both equations (−3.77 and −7.49 for equations (1) and (2), respectively) were significantly different at more than 99% confidence (p < 0.0001, t = 15.9 and degrees of freedom = 51). Lastly, given the large difference in sample size between both datasets, we used a Monte Carlo approach to estimate that the probability of having a regression slope similar to the one of the 0–5 day period (−7.49, equation 2) given a subsample of the pre-storm dataset was ~10% (Supplementary Material and Fig. S8).

### World shelf areas under TC influence

Using bathymetric data at a 1/4 degree resolution^37^, we calculated the total shelf area (depths less than 200 m) affected by TCs to be 8.5 × 10^6^ km^2^ (areas influenced by TCs taken from a previous study^79^). We tested and confirmed our calculation approach by comparing the global shelf area we obtained (27.9 × 10^6^ km^2^) with other estimates (26.2 × 10^6^ km^2^ and 27.7 × 10^6^ km^2^)^80,81^. To calculate the total area of river plumes in regions affected by TCs, we used data published in Table 3 of Kang *et al*.^80^. We considered the six major river systems in TC-affected regions: Mississippi, Orinoco, Ganges and Brahmaputra, Irrawaddy and Salween, Yangtze and Pearl. Averaging the river plume area during the six months of the TC-season for each of these six systems, the total combined plume area was 1.3 × 10^6^ km^2^. The latter represented a 15.3% of the total area of shelves affected by TC (8.5 × 10^6^ km^2^). Both areas were calculated using the 200 m isobaths as reference, but we could argue that using 50 m for both calculations would likely maintain the same relative relationship of 15%.

## Electronic supplementary material

Supplementary Material

## Data Availability

The datasets generated during and/or analysed during the current study are available from the corresponding author on reasonable request.
