# Supplementary material for: Contribution of hurricane-induced sediment resuspension to coastal oxygen dynamics
Source: Sci Rep. 2018 Oct 24;8:15740. doi: 10.1038/s41598-018-33640-3 (PMC6200824; doi:10.1038/s41598-018-33640-3)

**Contribution of hurricane-induced sediment resuspension to coastal oxygen dynamics**

**Supplementary Material**

**Authors:** Laura Bianucci*, Karthik Balaguru, Richard W. Smith, L. Ruby Leung, and Julia M. Moriarty

*corresponding author: [laura.bianucci@dfo-mpo.gc.ca](mailto:laura.bianucci@dfo-mpo.gc.ca)

***Sea surface salinity (SSS) and rainfall for Hurricanes Katrina and Rita***

We used the same data sources as for the SSS and rainfall composites in Figures 2c and d to look closely at the responses after Hurricanes Katrina and Rita (Fig. S1). Katrina made landfall in August 29 2005, very close to 29^o^N and 90^o^W (the location of our rain and SSS composites in Fig. 2). The peak rainfall (92 mm/d) generated an immediate response in SSS. After ~7 days of a SSS relaxation (i.e., it started increasing from its lowest value in August 30), it decreased again for approximately a week. The latter decrease was not attributed to rainfall, which was near zero. The SSS response to Rita presented a similar pattern, with a sharp freshening that lasted ~3 days right after the peak in precipitation (102 mm/d); again in this case, a second SSS decrease some days after a brief relaxation period was unassociated to rainfall. We attributed the surface freshening observed approximately a week after the precipitation peaks to the arrival of fresh water from the Mississippi River plume. This finding helped to establish our hypothesis, namely that it takes several days for the plume to arrive at the shelf after a hurricane event, allowing us to distinguish between processes directly associated to hurricanes and those influenced by the plume.

***Mississippi River plume timing and magnitude***

To further support our findings on the timing of the arrival of the Mississippi River (MR) plume and the magnitude of freshwater delivery, we examined flow data from the MR at Tarbert Landing (Fig. S2a) during a selected set of hurricanes that span a wide range of wind strength at landfall, landfall dates (month of landfall) and locations along the Gulf of Mexico coastline, and diverse paths over the MR watershed after landfall: Hurricanes Lili, Katrina, Rita, Audrey, Carla, Gilbert, Camille, Ike, and Gustav. The MR hydrograph of each hurricane was averaged in 5-day increments, including 5-10 d before landfall, 0-5 d before, 5-10 d after, etc. up to 25-30 d after (Fig. S2b). Average flow of the MR during each 5-day increment was then averaged for all hurricanes to produce a composite flow chart (Fig. S2c). Average flow peaked during the 15-20 day period, representing a 14% increase over pre-landfall conditions. The delay in the pulse reflects the duration of both the storm’s path over the watershed and the residence time of freshwater in the river.

Furthermore, we also analyzed flow rates from the USGS station at Belle Chasse, Louisiana (see location in Fig. S2a). We found that flow rates observed during hurricane events (~10,000 m^3^/s) were comparable to those during typical low to moderate flow periods reported previously^1^. The average water velocity corresponding to those flow periods is about 0.5 m/s^1^. For instance, during Hurricane Isaac in August 2012, we computed the average water velocity over the 3-day period 28 – 31 August to be 0.38 m/s. Considering that the distance between the USGS station at Belle Chasse and the Head of Passes is about 120 river kilometers^1^, we estimate the travel time to be nearly 3 days. Moreover, considering the travel time upstream of Belle Chasse and downstream from the Head of Passes to the river mouth, the total travel time for the sediments dissolved in the Mississippi River to reach the Northern Gulf of Mexico could be substantially longer.

***Numerical model of dissolved oxygen (DO) in the northern Gulf of Mexico: role of vertical diffusion, lateral advection and resuspension during Hurricane Humberto***

Coupled numerical models are often used to consider the relative roles of various processes in coastal systems. Models are especially useful for understanding dynamics in the bottom boundary layer and bottom water column, where hypoxia typically develops, because they can account for factors such as temperature, waves, currents, stratification, and sediment transport processes including resuspension, as well as biogeochemical processes^2,3,4,5^. Previous papers focusing on the Northern Gulf of Mexico, for example, have used models to consider the role of physical, sedimentary, and biogeochemical processes on oxygen dynamics in the bottom water column, as well as sediment transport^2,6,7^.

To consider the role of lateral versus vertical transport processes on DO concentrations in the Northern Gulf of Mexico, model output was analyzed for Hurricane Humberto, which hit the Texas-Louisiana coast on September 13, 2007. The output was from a coupled hydrodynamic-sediment transport-biogeochemical model developed with the Regional Ocean Modeling System (ROMS^8,9,10^), and was chosen because it accounts for resuspension of particulate organic matter, as well as other processes^11,12^. The full implementation for the Northern Gulf of Mexico was detailed in elsewhere^10,11^ and was based on previous published studies^6,7,10,14,15^. The coupled model that accounts for resuspension was previously used to analyze the role of resuspension during non-hurricane summertime conditions^12^, but was now analyzed for September 1-30, 2007, a time period that includes Hurricane Humberto. We considered two simulations: (1) a “Standard” model run with resuspension and (2) a “No-resuspension” sensitivity test. The No-resuspension model run was identical to the Standard simulation, except that seabed resuspension was prevented from occurring. Analysis focused on the western region of the Louisiana shelf, which was closest to the path of the hurricane (Fig. S3a).

A comparison of simulations with and without sediment resuspension showed the important role of this process on bottom DO. Resuspension increased the amount of particulate organic carbon (POC) in suspension (Fig. S3b) and decreased bottom DO (Fig. S3c) around the time of the hurricane (the passage of the storm is manifested by the peak in bed stress in September 13^th^ in Fig. S3d). Moreover, bottom DO was overall lower well after the hurricane in the Standard simulation, compared to the No-resuspension simulation (Fig. S3c). Furthermore, model results indicated that DO concentrations in the bottom water column are primarily a balance between vertical processes and biogeochemical DO consumption (Fig. S4a); in particular, the consumption is driven by remineralization of resuspended sediments (Fig. S4b). Note that vertical diffusion and advection were combined in Fig. S4a, but vertical diffusion accounted for an average of 82% with a standard deviation of 13% of this transport in the model during September 2007. Overall, vertical fluxes were ~15 times larger than horizontal fluxes in the region west of Atchafalaya Bay during September 2007. This result indicates that it is reasonable to neglect lateral fluxes in this region in September 2007, including during the passage of Hurricane Humberto.

Lastly, we can compute the amounts of DO ventilated and consumed by resuspended sediments in the model during 5 days following Hurricane Humberto (i.e., ΔDO_ventilation_ and ΔDO_sedim_ in the main text). We find that the modelled ΔDO_sedim_ is 56% of ΔDO_ventilation_ for this specific hurricane, in contrast with the 21% we calculated from the observations from multiple storms. The model results support our conclusions that, despite the uncertainty due to the limited availability of observations, the hurricane-induced resuspension of sediments plays a role in DO dynamics.

***Ability of hurricanes to homogenize the water column***

In order to evaluate the effect of hurricanes and tropical storms on the stratification of the northern Gulf of Mexico, we used temperature, salinity, and bathymetry information from data-assimilative HYbrid Coordinate Ocean Model (HYCOM, https://hycom.org/)^16^. From these data, we calculated density (ρ) and Δρ (ρ_bottom_ – ρ_surface_). Then, we found all hurricane tracks (wind speed > 33 m/s) and tropical storm tracks (wind speed between 17 and 33 m/s) north of 28.5^o^N and obtained an average of Δρ over a 2 by 2 degree box at each location, both before and after the storms (five days before and one day after). Since sediment resuspension occurs only over shallow waters, we masked those boxes to only include waters shallower than 50 m in the averaging of Δρ. Assuming that a Δρ ≤ 0.125 kg/m^3^ indicates a homogeneous water column^17^, the histogram of Δρ pre- and post-hurricanes and tropical storms (Fig. S5) showed that most (>70%) of the shallow waters under storms became homogeneous in their aftermath (red and yellow histograms in Fig. S5 for hurricanes and tropical storms, respectively).

***Dissolved oxygen data distribution***

The locations of the in situ stations that matched the three criteria explained in Methods are shown in Figure S6a, c, and e. The accompanying histograms (Fig. S6 b, d, and f) describe the typical dissolved oxygen (DO) conditions of each of the three periods: 1 to 14 days before hurricanes, 0 to 5 days after hurricanes and 5 to 15 days after hurricanes. From these histograms, we can appreciate that right after the hurricanes, there is a large increase in the number of DO observations with concentrations near saturation (between 4.4 and 4.8 mL/L), as well as a decrease in observations with DO < 2 mL/L with respect to pre-storm conditions . The latter indicates the alleviation of hypoxia due to ventilation. Furthermore, the DO histogram for the period 5 to 15 days after the hurricanes resembles the shape of the histogram for the pre-storm conditions, but with more observations with concentrations above 5 mL/L. The higher DO levels could be provided by phytoplankton blooms, which take advantage of the nutrients supplied by mixing and the river plume, as well as the post-storm restratification.

***Ventilation vs. sediment resuspension: Analysis of DO regressions***

In order to analyze the mechanisms driving bottom DO (DO_b_) concentrations from the pre-storm conditions to the period shortly after the hurricanes, we calculated the ordinary least square regressions of DO_b_ vs. vertical stratification (equations 1 and 2 in the main text, here with standard deviations shown in slopes and intercepts):

$$\begin{matrix} {DO}_{b}^{pre}=-\left( 3.77\pm0.73 \right){\cdot(\Delta\rho/\Delta z)}^{pre}+\left( 2.6\pm0.3 \right) & \left( S1 \right) \end{matrix}$$

$$\begin{matrix} {DO}_{b}^{post}=-\left( 7.49\pm0.55 \right){\cdot(\Delta\rho/\Delta z)}^{post}+\left( 4.2\pm0.2 \right) & \left( S2 \right) \end{matrix}$$

A schematic (Fig. S7 here and Fig. 3c in main text) represents graphically the steps taken to calculate the roles of hurricane-induced mixing (ΔDO_ventilation_) and the combined effects of re-stratification and DO consumption by the remineralization of resuspended sediments (ΔDO_sedim+restrat_). The calculations show the propagation^18^ of standard errors, which take into account the sample sizes (standard error = standard deviation/√N). To compute ΔDO_ventilation_, we started at the mean DO_b_ concentrations for the pre-hurricane period (equivalent to the mean stratification for the period, Δρ/Δz=0.33 kg/m^4^, applied to equation 1); then, we assumed a complete homogenization of the water column following the regression for the pre-storm conditions (red arrow in Fig. S7). Therefore, the ΔDO_ventilation_ was calculated as

ΔDO_ventilation_ = DO_b_^pre^(Δρ/Δz=0) – DO_b_^pre^(Δρ/Δz=0.33) = 2.6 – 1.4 = 1.2 ± 0.2 mL/L (S3)

Then, we proposed that ΔDO_sedim+restrat_ was the DO_b_ change from the homogenous conditions to the final mean conditions for the post-hurricane period (blue arrow in Fig. S7). Note that the final mean conditions are equivalent to applying the mean stratification for the 0 – 5 day period after the hurricanes (Δρ/Δz=0.28 kg/m^4^) to equation (2).

ΔDO_sedim+restrat_ = DO_b_^post^(Δρ/Δz=0.28) – DO_b_^pre^(Δρ/Δz=0) = 2.1 – 2.6 = –0.5 ± 0.4 mL/L (S4)

Therefore, by assuming that hurricanes achieve an immediate (but short-lived) homogenization of the water column, results in equations (S3) and (S4) indicate that ΔDO_sedim+restrat_ represents 42% of ΔDO_ventilation_ (0.5/1.2 = 0.42):

ΔDO_sedim+restrat_ = (0.42 ± 0.31) ΔDO_ventilation_ (S5)

The next step was to separate the effects of sediments and re-stratification from ΔDO_sedim+restrat_. Assuming that the availability of resuspended sediments increases DO_b_ consumption by 99% (± 8%) for any given change in stratification (see main text), we proposed to rewrite ΔDO_sedim+restrat_ as

ΔDO_sedim+restrat_ = ΔDO_sedim_ + ΔDO_restrat_ = (1.99 ± 0.08) ΔDO_restrat_ (S6)

Merging equations (S5) and (S6),

ΔDO_restrat_  = 0.42/1.99 ΔDO_ventilation_ = (0.21 ± 0.15) ΔDO_ventilation_ (S7)

ΔDO_sedim_ = 0.99 ΔDO_restrat_ = (0.21 ± 0.16) ΔDO_ventilation_ (S8)

Then, we concluded that the effect of the consumption of DO_b_ by the remineralization of resuspended sediments, as well as the effect of re-stratification, represent up to21% of the re-aeration of the water column (although with large uncertainty).

As a side note regarding the post-storm period, it could be argued that lower DO_b_ levels could be attained in the absence of resuspended sediments just by longer exposure to stratified conditions. While we cannot fully assess the time-exposure to stratification with our dataset, we can use the time elapsed since the hurricanes as a good proxy. Our post-storm data show that DO_b_ is not necessarily lower at longer time-exposure since hurricanes. For instance, DO_b_ is the highest at 3.3 days from the storm (Hurricane Cindy in this case), which is the second longest exposure period in our post-storm dataset.

***Dealing with datasets with different sample sizes: Monte Carlo approach***

When comparing the pre-storm dataset against the observations right after the hurricanes (0 – 5 days), we dealt with two very different sample sizes: 48 profiles for the period before and only 7 profiles for the period after the hurricanes. To test the probability of having a regression slope similar to the one of the 0 – 5 day period (–7.49, equation (2) in the main text) given a subsample of the pre-storm dataset, we followed a Monte Carlo approach. We took 10,000 random subsamples of 7 profiles out of the 48 available for the pre-hurricane period and created a probability distribution function for the slope of the regressions of each of those 10,000 subsamples (Fig. S8). The mean slope of the distribution (–4.45, dashed black line) was close to the slope of the full pre-hurricane dataset (–3.77, blue line). The probability of having a regression slope ≤ –7.49 (dot-dashed red line) was ~10%; furthermore, the latter slope was more than 1 standard deviation away from the mean of the 10,000 subsampled pre-storm slopes. These results support the argument that the differences in the slopes where not due to the different size of the pre- and post-storm datasets.

***Bottom oxygen vs. stratification regressions: model I and model II calculations***

So far, we described the relationship between DO­­­_b_ and Δρ/Δz using linear (ordinary) least squares in equations 1, 2 (main text), S1 and S2 (here in the supplement). There are two main assumptions in this model I least square fit: a) “X” (Δρ/Δz) is independent while “Y” (DO­_b_) is the dependent variable, i.e. X drives or controls Y; and b) X has little or no measurement error. The former is a good assumption for our case (stratification has already been shown to be an important predictor of DO_b_ in the Gulf of Mexico^10,19^). In terms of errors in Δρ/Δz, while it is likely that the measurement errors associated with temperature and salinity (used to calculate ρ) are much smaller than the measurement errors in DO_b_, we may not be able to neglect them. By applying this linear fit and assuming no error in Δρ/Δz, the resulting slopes may be underestimated. In contrast, model II regression methods account explicitly for errors in X. However, they usually consider a symmetric relationship between X and Y, i.e. X vs. Y is the same as Y vs. X. In other words, neither variable is independent, but both are assumed to be dependent on some other (likely unknown) variable. We followed the advice of a relatively recent study^20^, which argues that authors tend to prioritize the presence or absence of error to decide their fitting method, while the symmetry-asymmetry issue should be the criterion instead because it addresses the relationship between variables. Nevertheless, we computed the regressions with two model II least square fits (Reduced Major Axis or Geometric Mean^21^ and Bisector^22^) and present them in Table S1. The slopes of the pre- and post-storm datasets are significantly different with any of these methods and the final results still support our main conclusions, even if with slightly different numbers: the sediment resuspension as a fraction of re-aeration is 21% for ordinary least squares, 12% for the Geometric Mean least squares, and 16% for Bisector least squares.

***Re-establishment of pre-storm conditions****:* ***The return of hypoxia***

We used the relationship between bottom DO and stratification to investigate the role of the processes at play during the 5 to 15 day period after the hurricanes (Fig. S9a):

$$\begin{matrix} {DO}_{b}^{after 5-15}=-2.99{\cdot(\Delta\rho/\Delta z)}^{after 5-15}+3.3 & \left( S9 \right) \end{matrix}$$

with R^2^ = 0.72 (N = 36). While both the R values and slope in equation (S9) were statistically different to those in equations (1) and (2) in the main text, the slope was much closer to the pre-storm conditions (equation 1). This similarity in slope, plus the fact that the intercept in equation (S9) was higher than in equation (1) (3.3 vs. 2.6 mL/L), made the regression for the 5 – 15 days post-storm period appear similar to the one for the pre-storm conditions, but at a higher background concentration of bottom DO (Fig. S9a, blue vs. black lines). We interpreted this result as re-establishment of the processes active before the storm, but in a water column re-oxygenated by hurricane-induced ventilation.

The stations with bottom hypoxia in the period 5 – 15 days after hurricanes presented strong stratification (Δρ/Δz > 0.6 kg/m^4^, Fig. S9a and b); furthermore, the buildup of this stratification was due to high vertical gradients in salinity rather than temperature gradients (Fig. S9b, with ΔS/Δz and ΔT/Δz calculated analogously to Δρ/Δz). This result implies that the river plume influenced these stations, since the plume is a shallow feature in the region (with thickness from a few meters to less than 10 m^23,24^) and generates large vertical salinity gradients. Therefore, we concluded that the buildup of strong stratification by the arrival of the hurricane-enhanced plume (even stronger than during pre-storm conditions) was a key driver for the re-establishment of hypoxic levels. Furthermore, the plume likely contributed more organic matter to be remineralized, as suggested by the further increase in TSM the week after the hurricanes (Fig. 2b).

***Total Suspended Matter in regions without large river plumes***

Sediment resuspension is not limited to deltaic regions of the shelf in the Gulf of Mexico; mobile sediments extend across all regions of the Gulf shelf^25,26^, and modeling results suggest hurricane-induced sediment erosion occurs over large areas^6,27^. Moreover, regions without the influence of large rivers can also show high TSM concentrations after the passage of a hurricane^28^. Here, using satellite-derived TSM data^29,30^, we show an example for the East Florida shelf during Hurricane Jeanne in September 2004 (Fig. S10). Furthermore, non-deltaic continental shelf sediments are still largely fine-grained and available for resuspension, and as mentioned in the main text, they contain about double the percentage organic carbon as deltaic sediments^31^. Therefore, even if the mass of resuspended non-deltaic sediments may be smaller than resuspension in deltaic regions, their decomposition can still have an effect on DO dynamics.

**References**

1. Nittrouer, J. A., Shaw, J., Lamb, M. P., & Mohrig, D. Spatial and temporal trends for water-flow velocity and bed-material sediment transport in the lower Mississippi River. *Geological Society of America Bulletin* **124**, 3-4, doi:10.1130/B30497.1 (2012).
2. Fennel, Katja, et al. Effects of model physics on hypoxia simulations for the northern Gulf of Mexico: A model intercomparison. *Journal of Geophysical Research: Oceans* **121(8)**, 5731-5750, doi: 10.1002/2015JC011577 (2016).
3. Bonaldo D. et al. Interactions among Adriatic continental margin morphology, deep circulation and bedform patterns. *Marine Geology* **375**, 82-98, doi: 10.1016/j.margeo.2015.09.012 (2016).
4. Carniel S. et al. Off-shelf fluxes across the southern Adriatic margin: factors controlling dense water-driven-transport phenomena. *Marine Geology* **375**, 44-63, doi: 10.1016/j.margeo.2015.08.016 (2016).

Warner, J. C., Armstrong, B., He, R. & Zambon, J.B. Development of a coupled ocean-atmosphere-wave-sediment transport (COAWST) modeling system. *Ocean Modelling* **35**, 230-244 (2010).

Xu, K. *et al.* Shelf sediment transport during hurricanes Katrina and Rita. *Computers & Geosciences* **90**, 24-39, doi:10.1016/j.cageo.2015.10.009 (2016)

Yu, L., Fennel, K. & Laurent, A. A modeling study of physical controls on hypoxia generation in the northern Gulf of Mexico. *Journal of Geophysical Research-Oceans* **120**, 5019-5039, doi:10.1002/2014JC010634 (2015).

1. Haidvogel, D.B., Arango, H. G., Hedstrom, K., Beckmann, A., Malanotte-Rizzoli, P., & Shchepetkin, A. F. Model evaluation experiments in the North Atlantic Basin: simulations in nonlinear terrain-following coordinates. *Dynamics of Atmospheres and Oceans* **32**, 239-281 (2000).
2. Warner, J. C., Sherwood, C. R., Signell, R.P., Harris, C.K., & Arango, H.G. Development of a three-diminesional, regional, coupled wave, current, and sediment-transport model. *Computers & Geosciences* **34**, 1284-1306 (2008).
3. Fennel, K., Hu, J., Laurent, A., Marta-Almeida, M. & Hetland, R. Sensitivity of hypoxia predictions for the northern Gulf of Mexico to sediment oxygen consumption and model nesting. *Journal of Geophysical Research-Oceans* **118**, 990-1002, doi:10.1002/jgrc.20077 (2013).

Moriarty, J. M. *et al.* The roles of resuspension, diffusion and biogeochemical processes on oxygen dynamics offshore of the Rhône River, France: a numerical modeling study. *Biogeosciences* **14**, 1919-1946, doi:10.5194/bg-14-1919-2017 (2017).

1. Moriarty, J.M., Harris, C.K., Friedrichs, M.A.M., Fennel, K., & Xu, K. Impact of seabed resuspension on oxygen and nitrogen dynamics in the northern Gulf of Mexico: A numerical modeling study. Published online at *Journal of Geophysical Research-Oceans*, doi: 10.1029/2018JC013950 (2018).
2. Moriarty, J. M. The Role of Seabed Resuspension on Oxygen and Nutrient Dynamics in Coastal Systems: A Numerical Modeling Study Ph.D. thesis, Virginia Institute of Marine Science, (2017).
3. Xu, K., Harris, C.K., Hetland, R.D., & Kaihatu, J. M. Dispersal of Misssissippi and Atchafalaya sediment on the Texas-Louisiana shelf: Model estimates for the year 1993. *Continental Shelf Research* **31**, 1558-1575 (2011).

Hetland, R. D. & DiMarco, S. F. Skill assessment of a hydrodynamic model of circulation over the Texas-Louisiana continental shelf. *Ocean Modeling* **43-44**, 64-76 (2012).

1. Chassignet, E. P. et al. The HYCOM (HYbrid Coordinate Ocean Model) data assimilative system. *Journal of Marine Systems* **65**, 60-83, doi:10.1016/j.jmarsys.2005.09.016 (2007).
2. Levitus S. Climatological Atlas of the World Ocean. Princeton, NJ: NOAA/ERL GFDL Professional Paper 13, NTIS PB83-184093 (1982).
3. Ku, H. H. Notes on the use of propagation of error formulas. *Journal of Research of the National Bureau of Standards* 70C (4): 263-273, doi:10.6028/jres.070c.025 (1966).
4. Wiseman, W., Rabalais, N., Turner, R., Dinnel, S. & MacNaughton, A. Seasonal and interannual variability within the Louisiana coastal current: stratification and hypoxia. *Journal of Marine Systems* **12**, 237-248, doi:10.1016/S0924-7963(96)00100-5 (1997).
5. Smith, R. Use and Misuse of the Reduced Major Axis for Line-Fitting. *American Journal of Physical Anthropology* **140**, 476-486, doi:10.1002/ajpa.21090 (2009).
6. Ricker, W.E. Linear regressions in Fishery Research. *Journal of the Fisheries Research Board of Canada* 30: 409-434, doi: 10.1139/f73-072 (1973).
7. Sprent, P. & Dolby, G.R. The Geometric Mean Functional Relationship. *Biometrics* **36**, 547-550, doi:10.2307/2530224 (1980).
8. Dagg, M. J., Ammerman, J. W., Amon, R. M., Gardner, W. S., Green, R. E., & Lohrenz, S. E. A review of water column processes influencing hypoxia in the northern Gulf of Mexico. *Estuaries and Coasts* **30**, 735-752, doi: 10.1007/BF02841331 (2007).
9. Zhang, X., Hetland, R. D., Marta-Almeida, M., & DiMarco, S. F. A numerical investigation of the Mississippi and Atchafalaya freshwater transport, filling and flushing times on the Texas-Louisiana Shelf. *Journal of Geophysical Research: Oceans*, **117,** C11009, doi:10.1029/2012JC008108 (2012).
10. Keen, T. R. & Slingerland, R. L. Four storm-event beds and the tropical cyclones that produced them; a numerical hindcast. *Journal of Sedimentary Research* **63**, 218-232, doi:10.1306/d4267ac8-2b26-11d7-8648000102c1865d (1993).
11. Williams, S.J., Arsenault, M.A., Buczkowski, B.J., Reid, J.A., Flocks, J.G., Kulp, M.A., Penland, S., & Jenkins, C.J. Surficial sediment character of the Louisiana offshore Continental Shelf region: a GIS compilation. U. S. Geological Survey Open-File Report 2006-1195 (2006). Online at http://pubs.usgs.gov/of/2006/1195/ index.htm
12. Hedges, J. I. & Keil, R. G. Sedimentary organic matter preservation: an assessment and speculative synthesis. *Marine Chemistry* **49**, 81-115, doi:10.1016/0304-4203(95)00008-F (1995).
13. Hu, C., & Muller-Karger, F. E. Response of sea surface properties to Hurricane Dennis in the eastern Gulf of Mexico. *Geophysical Research Letters* **34,** L07606, doi:10.1029/2006GL028935 (2007).
14. Fanton d'Andon, O. *et al.* in *2009 IEEE International Geoscience & Remote Sensing Symposium.* (IEEE Geoscience and Remote Sensing Society).
15. Maritorena, S., d'Andon, O. H. F., Mangin, A. & Siegel, D. A. Merged satellite ocean color data products using a bio-optical model: Characteristics, benefits and issues. *Remote Sensing of Environment* **114**, 1791-1804, doi:10.1016/j.rse.2010.04.002 (2010).
16. Bevington, P. R., & Robinson, D. K. *Data reduction and error analysis for the physical sciences* (McGraw-Hill, 1992).

**Tables**

**Table S1. Bottom dissolved oxygen (DO) vs. Stratification calculations with different regression models**

|  | Ordinary least squares (model I) | Geometric mean (model II) | Bisector  (model II) |
| --- | --- | --- | --- |
| **Pre-storm (N=48)** | | | |
| Slope^*^ (mL L^-1^ kg^-1^ m^4^) | –3.77 ± 0.73 | –6.23 ± 0.82 | –5.56 ± 0.78 |
| Intercept^*^ (mL L^-1^) | 2.63 ± 0.27 | 3.43 ± 0.30 | 3.21 ± 0.28 |
| **Post-storm (0-5 days; N=7)** | | | |
| Slope^*^ (mL L^-1^ kg^-1^ m^4^) | –7.49 ± 0.55 | –7.58 ± 0.55 | –7.58 ± 0.55 |
| Intercept^*^ (mL L^-1^) | 4.23 ± 0.17 | 4.26 ± 0.17 | 4.26 ± 0.17 |
| **T-test for pre- and post-storm slopes (degrees of freedom: 51)** | | | |
| t-values | –15.9 | –5.7 | –8.6 |
| p | <0.0001 | <0.0001 | <0.0001 |
| **Calculations^**^** | | | |
| Increase in DO_b_ consumption by resuspended sediments (slope_post_ / slope_pre_) | 1.99 ± 0.08 | 1.22 ± 0.04 | 1.37 ± 0.05 |
| ΔDO_ventilation_ (mL L^-1^) | 1.22 ± 0.15 | 2.02 ± 0.15 | 1.81 ± 0.15 |
| ΔDO_sedim+restart_ (mL L^-1^) | –0.51 ± 0.38 | –1.30 ± 0.38 | –1.09 ± 0.38 |
| ΔDO_sedim+restrat_ /ΔDO_ventilation_ | 0.42 ± 0.31 | 0.64 ± 0.19 | 0.60 ± 0.21 |
| ΔDO_restrat_ / ΔDO_ventilation_ | 0.21 ± 0.16 | 0.53 ± 0.16 | 0.44 ± 0.16 |
| ΔDO_sedim_ / ΔDO_ventilation_ | 0.21 ± 0.15 | 0.12 ± 0.03 | 0.16 ± 0.06 |

^*^Uncertainties given as standard deviations^12^. For model II regressions, they are based on the symmetrical limits for a model I regression following Ricker's treatment^6^ and equations from Bevington and Robinson^12^.

^**^Calculations propagate standard errors in order to take into account the different sample sizes.

**Figures**

**Figure S1. Rainfall and sea surface salinity (SSS) response to Hurricanes Katrina and Rita.** (Top) Time series of precipitation at 29^o^N and 90^o^W. (Bottom) Hövmoller diagram (latitude vs. time) for SSS at 90^o^W. Both X axes extend from August 20 to October 9 2005.


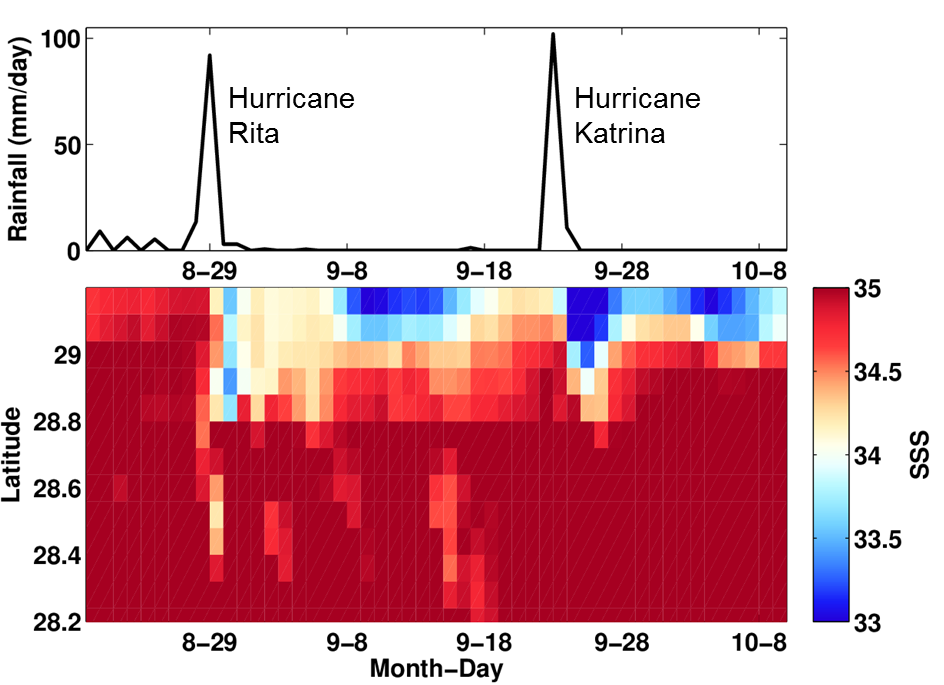


**Figure S2. Mississippi River hydrograph pre- and post-storm landfall at Tarbert Landing.** (a) Location of USGS hydrographs at Tarbert Landing and Belle Chasse. (b) 5 day averages of MR flow during 9 selected GOM hurricanes. Light blue background: pre-landfall, light red background: post-landfall. (c) Composite average.


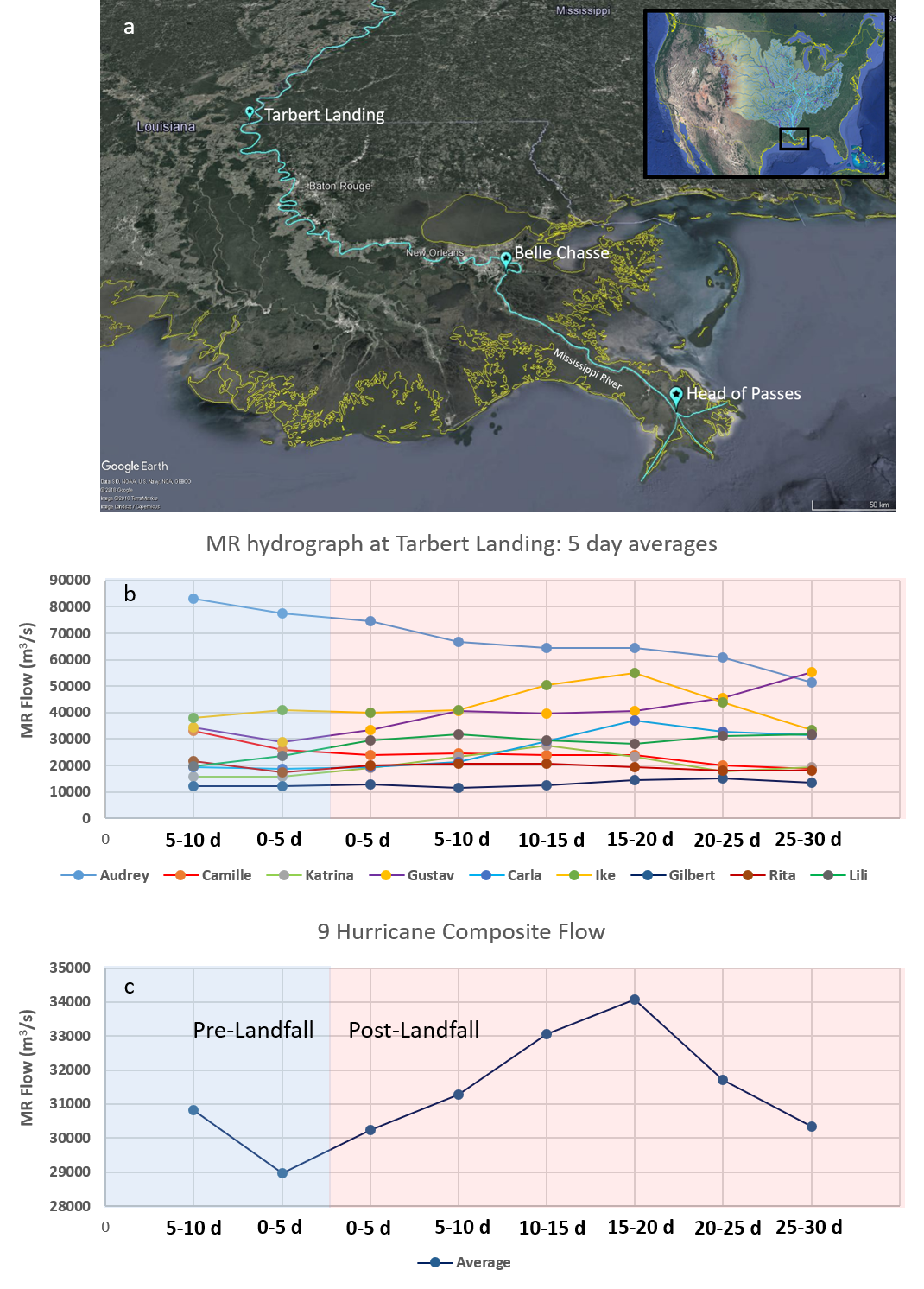


**Figure S3. Results from a numerical ocean model during Hurricane Humberto.** (a) The blue line shows the track of Hurricane Humberto (September 2007) and the blue shading indicates the region of analysis for ROMS model results. The time series in (b), (c) and (d) show median (bold lines) and 5^th^-95^th^ percentiles (shading) in the region of analysis for particulate organic carbon (POC), bottom DO, and bed stress, respectively. Standard (i.e. with resuspension) and No-resuspension simulations are in black and pink, respectively. The vertical dashed line indicates September 13^th^, when Hurricane Humberto made landfall.

**
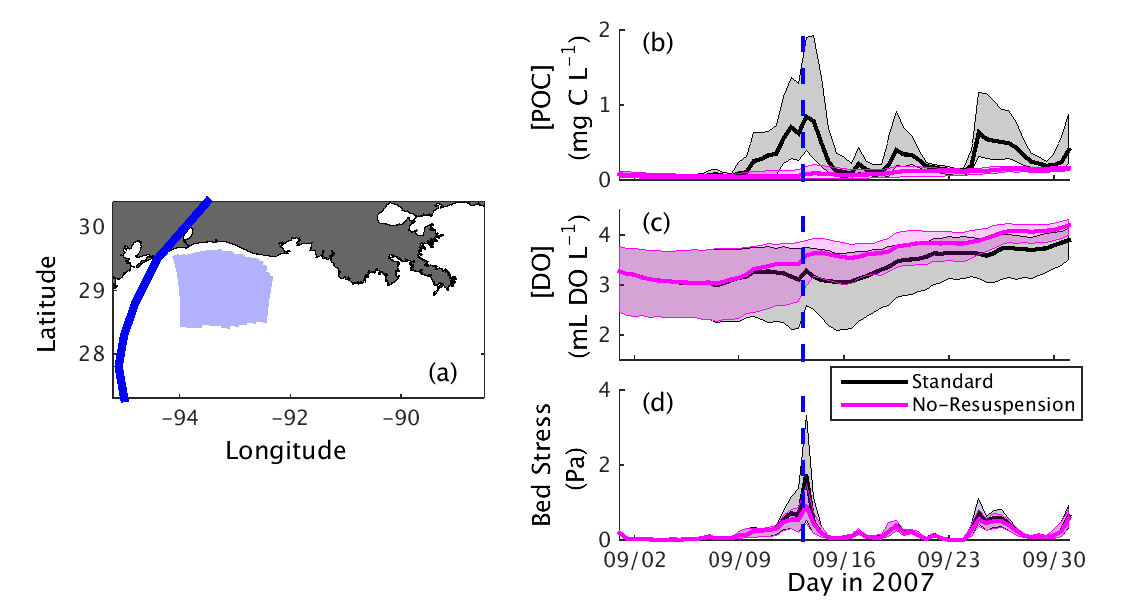
**

**Figure S4. Modelled changes in physical and biogeochemical processes during Hurricane Humberto.** (a) Time series of net change in bottom water DO, as well as changes due to biogeochemical (BGC) consumption, vertical transport (diffusion plus advection), and horizontal advection in the numerical model. (b) Fraction of DO consumption and vertical transport that is due to resuspension. These fractions were calculated by dividing the difference in values between the Standard and No-resuspension simulations by the value from the Standard simulation. All time-series are averages for the region indicated in Fig. S3a.

**
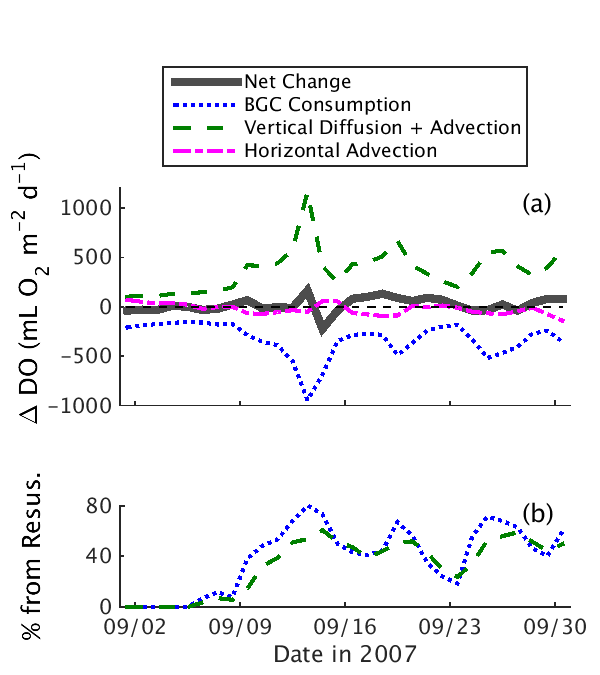
**

**Figure S5. Histograms of stratification pre- and post-storms.** Δρ from HYCOM five days before and one day after storms (hurricanes in left panel, tropical storms in right panel).

**
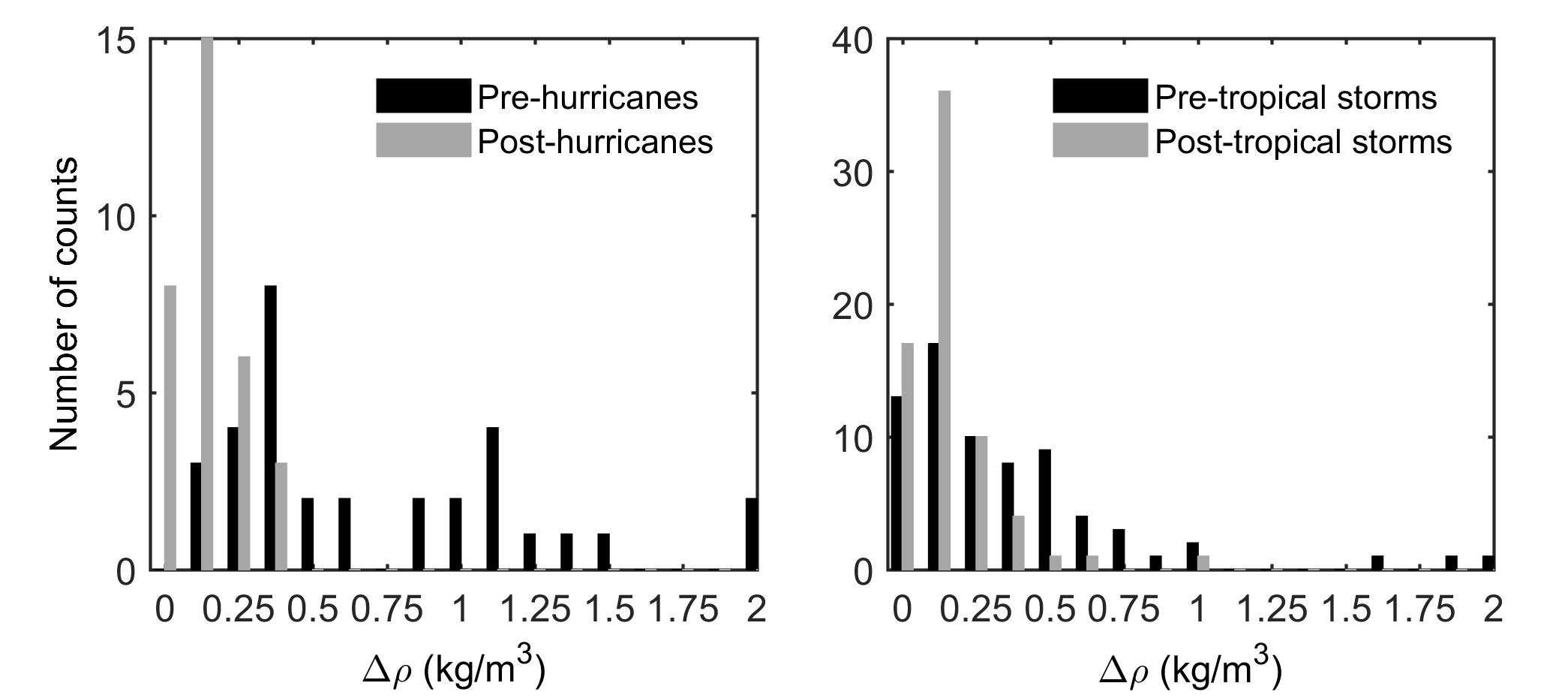
**

**Figure S6. Location and histograms of in situ dissolved oxygen (DO) data**. Location of in situ observations and corresponding histograms of DO for each of the three periods of analysis: (a, b) 4 to 14 days before hurricanes, (c, d) 0 to 5 days after hurricanes, and (e, f) 5 to 15 days after hurricanes. In (e), gray circles show the full dataset for the period while black circles show the spatially subsampled data that cover the same area as the observations for the 0 to 5 days period; (f) shows the histograms corresponding to the black circles.

**Figure S7. Schematic for 2-step dissolved oxygen (DO) analysis.** Gray and black lines represent the regressions for bottom DO vs. stratification for the pre-storm and post-storm (0 – 5 days) periods (equations (1) and (2) in the main text, respectively). Red arrow indicates the change in DO due purely to mixing and ventilation from the mean pre-storm conditions to complete homogenization (nil stratification). Blue arrow indicates the change in DO until the mean post-storm conditions due to the effect of both restratification after the hurricane’s passage and the enhanced DO consumption due to the remineralization of resuspended sediments. Horizontal dotted lines highlight the values of bottom DO for the pre-storm, post-storm, and fully-homogenized conditions.

**Figure S8. Probability distribution function of regression slopes of randomly subsampled data for the pre-storm period.** The full dataset for the pre-hurricane period had with 48 profiles; it was randomly subsampled 10,000 times, keeping only 7 profiles each time. Regression slopes are shown for the full dataset (blue line), the mean of the subsampled datasets (dashed black line), and the period 0 – 5 days after the hurricanes (dot-dashed red line).


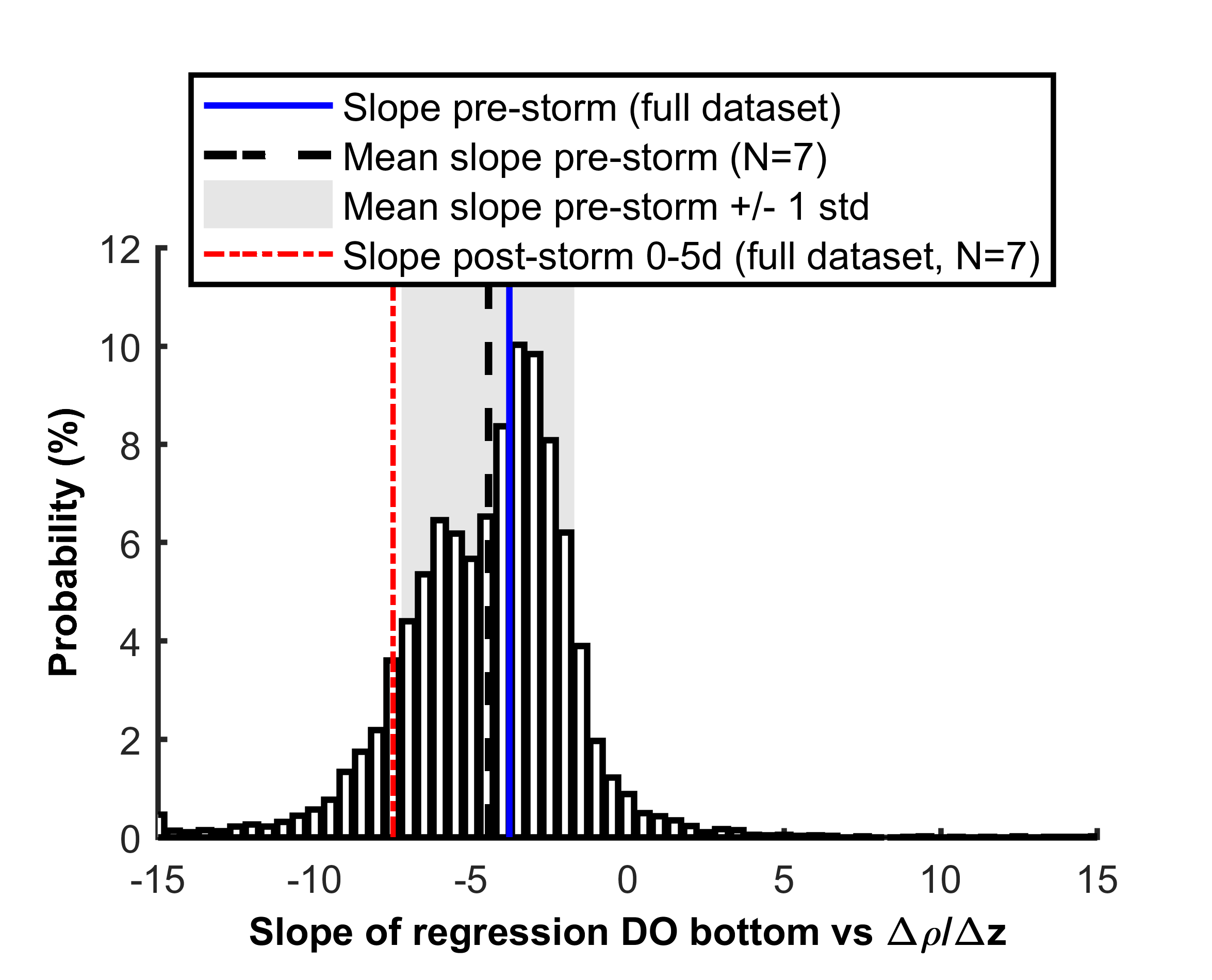


**Figure S9. Analysis of dissolved oxygen (DO) data for the period 5 to 15 days after hurricanes.** (a) Bottom DO vs. stratification as in Fig. 3b of main text, but adding period 5 – 15 days after hurricanes (blue triangles and blue regression line). The horizontal dotted line indicates the hypoxic threshold (1.4 mL/L). (b) Vertical gradients of salinity (left axes, circles) and temperature (right axes, diamonds) vs. stratification. Color scale represents bottom DO concentrations (the sharp change in colors occurs at 1.4 mL/L).

**
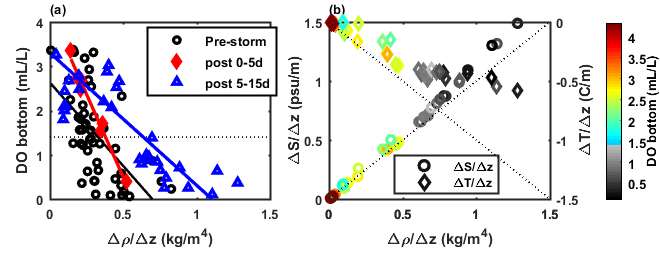
**

**Figure S10. Map of sediment resuspension in the US East Coast**. Weekly total suspended matter (TSM) at surface in the East Coast for the week in which Hurricane Jeanne made landfall (21 to 28 September 2004) and Jeanne’s track (numbers indicate day of September at midnight). Color scale is analogous to that of Fig. 1a in the main text; the 50, 100, and 200 m isobaths are shown as gray contours. Map was created using MATLAB (R2014a). TSM at 4 km spatial resolution was obtained from the European Space Agency’s GlobColour Project (www.globcolour.info); these data have been developed, validated, and distributed by ACRI-ST, France


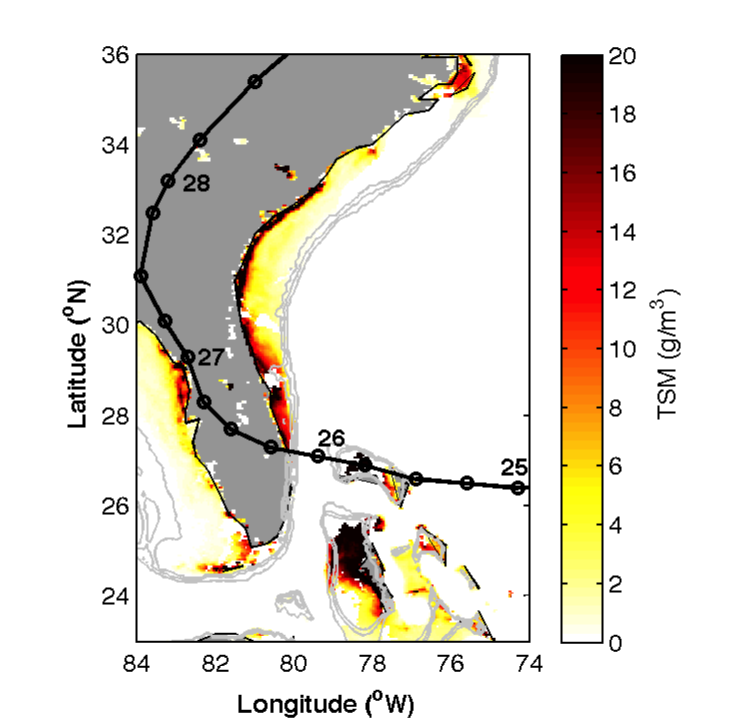

Supplement: Supplementary file 1 — Supplementary Material [file 41598_2018_33640_MOESM1_ESM.docx]
